# Effect of adjuvant radiotherapy after breast-conserving surgery in elder women with early-stage breast cancer: a propensity-score matching analysis

**DOI:** 10.3389/fonc.2023.1012139

**Published:** 2023-10-13

**Authors:** Tingting Han, Mingwei Shi, Qiwei Chen, Dongbo Chen, Jiqing Hao

**Affiliations:** ^1^ Department of Radiation Oncology, The First Affiliated Hospital of Anhui Medical University, Hefei, China; ^2^ Department of Oncology, Hefei BOE Hospital, Hefei, China; ^3^ Department of Oncology, The First Affiliated Hospital of Anhui Medical University, Hefei, China

**Keywords:** breast cancer, SEER database, breast-conserving surgery, adjuvant radiotherapy, propensity-score matching

## Abstract

**Purpose:**

The study aimed to explore the role of adjuvant radiotherapy (RT) after breast-conserving surgery (BCS) in elder women with early-stage breast cancer (BC).

**Methods:**

BC patients with 70-79 years of age, stage T1‐2N0‐1M0, undergoing BCS were screened in the Surveillance, Epidemiology and End Results (SEER) database between 2010 and 2015. The clinicopathological characteristics were balanced with propensity-score matching (PSM) method. Kaplan–Meier curves and Cox regression analyses were performed to determine the impact of adjuvant RT on BC patients.

**Results:**

Ultimately, 12,310 patients treated with adjuvant RT and 4837 patients treated with no RT, were involved in the analysis. Overall, patients treated with adjuvant RT was associated with a better breast cancer-specific survival (BCSS) (HR: 1.980 [1.596- 2.456], P < 0.001) and overall survival (OS) (HR: 2.214 [1.966- 2.494], P < 0.001) than those who did not undergo RT. After 1:1 PSM, adjuvant RT still performed advantage in both BCSS (HR: 1.918 [1.439- 2.557], P < 0.001) and OS (HR: 2.235 [1.904- 2.624], P < 0.001). In the multivariate COX analysis of BCSS, widowed, divorced and separated patients, tumor grade III, T2 stage, N1 stage, no RT, molecular subtypes with luminal B and triple negative were associated with a shorter BCSS (P < 0.05). In the multivariate COX analysis of OS, age ≥74 years, widowed, divorced and separated patients, tumor grade II/III, T2 stage, no RT, no chemotherapy, molecular subtypes with triple negative were associated with a shorter OS (P < 0.05). Furthermore, the advantages of adjuvant RT were observed in all subgroup analysis.

**Conclusion:**

Adjuvant RT after BCS can improve both BCSS and OS in elderly patients with early-stage BC. Additionally, all subgroups analysis-derived BCSS and OS were in support of RT.

## Introduction

Breast cancer (BC) is one of the commonest cancers in the United States, which is the second leading cause of cancer deaths among women. The incidence rate of BC has increased in women aged 70 years or older in recent years (1). For most early-stage BC patients, the standard treatment strategy is the performance of breast-conserving surgery (BCS) followed by adjuvant whole breast radiotherapy (RT) and adjuvant endocrine therapy (ET) based on hormone receptor status (2). In elderly patients, considering the increase in the proportion of hormone receptor-positive tumors and the lower biological invasiveness, treatment strategies tend to be conservative (1, 3).

Two large randomized clinical trials, CALGB9343 and PRIME II, support lower-risk elderly women with early-stage BC who are planning to receive ET with omission of RT after BCS (4, 5). CALGB9343 trial randomly selected 636 breast cancer patients aged 70 years, clinical stage T1N0M0, ER receptor positive, and divided into tamoxifen plus RT group and tamoxifen alone group after BCS. The long-range results with a 12.6 years median follow-up time showed that although there was a slight improvement in locoregional recurrence with the use of RT (10% vs. 2%), no significant differences were seen between the two groups in mastectomy time, distant metastasis time, breast cancer-specific survival (BCSS) or overall survival (OS) (4). Similarly, Prime II trail in the UK also verified the efficacy of omitting RT in a similar patient population, except that this study included 1,326 women aged 65 years and tumors up to 3 cm. After a 5 years median follow-up time, the results also supported that RT reduce the recurrence rate of ipsilateral breast tumors (1.3% vs 4.1%, P=0.0002) without difference in distant metastasis and regional recurrence (5). In addition, after the publication of the CALGB9343 study, the surveillance, epidemiology, and end result (SEER) registration were analyzed to evaluate practice patterns, showing statistically significant decrease in the frequency of RT delivery over time across age groups, tumor grading, and tumor size and regardless of laterality (6).

For elderly BC patients with early stage, treatment strategies tend to conduct the omission of RT in most cases. Although previous clinical trials have shown no survival benefit from radiotherapy in BC patients with clinical stage I (4, 5), and the reduction of using RT in practice patterns (6), they should be validated to determine their applicability to the general population in the reality setting. This article discusses the role of RT in early-stage BC patients in SEER database, and hopes to play a clinically guiding role.

## Materials and methods

### Study patients

The data for this retrospective study came from the SEER database, the largest public cancer database that collects cancer data from 18 population-based cancer registries, accounting for approximately 28% of the US population (1). This analysis was limited to 70-79 years female patients, and diagnosed with stage T1‐2, N0‐1, M0 early‐stage BC (AJCC stages I, IIA, part of IIB) between 2010 and 2015. We chose to exclude 80 years of age or older patients to reduce the risk of potentially confounding secondary deaths. For the study, patients must meet the criteria as following: positive pathological confirmation, invasive ductal carcinoma (ICD-0-3 code: 8500/3), performing breast-conserving surgery (surgery code: 20-24), only one primary tumor, tumor on one side, no radiotherapy or undergoing adjuvant radiotherapy. Patients with incomplete stage, unknown molecular subtype, unknown survival time and cause of death, and death within 1 month after surgery were excluded.

### Clinicopathological data

Patient demographic characteristics including age, marital status, race, grade, laterality, T stage, N stage, chemotherapy information, tumor molecular subtypes, treatment strategies and survivals data were obtained from the SEER database. A median age of 74 years was chosen as the cut-off value.

### Statistical analysis

The primary endpoint of this study was BCSS and OS. Chi-square test was performed to compare the baseline characteristics of the two groups. Survival curves were generated by Kaplan-Meier estimation. The difference of survival curves was compared using log-rank test. The matching of patients was analyzed by propensity-score matching (PSM) method, and the caliper value was set to 0.001. The propensity score distributions were evaluated graphically for common support, while the balance between the two groups was assessed by computing the standardized mean difference of the covariates (7). Univariate and multivariate COX regression analysis were performed to assess mortality risk and conduct subgroup analysis. All statistical analyses were conducted with IBM SPSS Statistics software version 24.0, and R software version 3.6.2. A statistically significant value was set as P<0.05.

## Results

### Patient demographics

17,147 patients were included totally in this retrospective cohort study. The patients were divided into two groups, of which 12,310 patients received breast-conserving adjuvant radiotherapy (RT group) and 4837 patients did not receive radiotherapy (No RT group). The two groups had significant differences in age, marital status, race, tumor grade, N stage, chemotherapy information, and molecular subtypes (**Table 1**). In particular, the radiotherapy group has a higher percentage of younger patients, married or divorced patients, white patients, highly differentiated tumors, N1 patients, undergoing chemotherapy, and molecular subtypes of triple negative or luminal A. This indicates that the characteristics of imbalanced baseline were existed between the two groups. After 1:1 PSM, a total of 4610 pairs patients were successfully matched (**Table 1**). The propensity score distributions between the two groups following PSM exhibited nearly ideal common support (**Figure S1**). Additionally, the covariates were well-balanced between the two treatment groups after PSM, with standardized mean differences below 5% for all covariates (**Figure S2**). In the matching model, the baseline characteristics including age, marital status, race, tumor grade, laterality, T stage, N stage, chemotherapy information, and molecular subtype are balanced. Because Grade IV did not match successfully in the two groups and the sample size was too small, in order to prevent bias, the Grade IV information was deleted in the subsequent calculations.

**Table 1:** The clinical and pathological characteristics for patients before and after PSM.

Variables	Initial cohort			PSM cohort		
	RT (%)	No RT (%)	P value	RT (%)	No RT	P value
**Age**			< 0.001			0.660
<74	6346 (74.7)	2147 (25.3)		2029 (49.7)	2050 (50.3)	
≥74	5964 (68.9)	2690 (31.1)		2581 (50.2)	2560 (49.8)	
**Marital status**			< 0.001			0.913
Married/Partner	6556 (74.4)	2260 (25.6)		2201 (50.1)	2190 (49.9)	
Single	974 (68.6)	446 (31.4)		400 (49.0)	417 (51.0)	
Widowed/Divorced/Separated	4213 (69.8)	1824 (30.2)		1760 (50.2)	1747 (49.8)	
Unknown	567 (64.9)	307 (35.1)		249 (49.3)	256 (50.7)	
**Race**			0.012			0.527
White	10513 (72.2)	4044 (27.8)		3931 (50.0)	3936 (50.0)	
Black	950 (69.2)	423 (30.8)		346 (48.7)	364 (51.3)	
Others	847 (69.6)	370 (30.4)		333 (51.8)	310 (48.2)	
**Grade**			< 0.001			0.855
I	3713 (69.2)	1651 (30.8)		1622 (50.0)	1624 (50.0)	
II	5551 (73.0)	2049 (27.0)		2007 (50.3)	1986 (49.7)	
III	2833 (73.2)	1036 (26.8)		914 (49.5)	931 (50.5)	
IV	11 (84.6)	2 (15.4)		1 (100.0)	0 (0.0)	
Unknown	202 (67.1)	99 (32.9)		66 (48.9)	69 (51.1)	
**Laterality**			0.081			1
Right	6074 (72.4)	2315 (27.6)		2206 (50.0)	2206 (50.0)	
center	6236 (71.2)	2522 (28.8)		2404 (50.0)	2404 (50.0)	
**T stage**			0.894			0.866
T1	10137 (71.8)	3979 (28.2)		3853 (50.0)	3859 (50.0)	
T2	2173 (71.7)	858 (28.3)		757 (50.2)	751 (49.8)	
**N stage**			< 0.001			0.813
N0	10510 (71.1)	4281 (28.9)		4117 (50.0)	4124 (50.0)	
N1	1800 (76.4)	556 (23.6)		493 (50.4)	486 (49.6)	
**Chemotherapy**			< 0.001			0.592
Yes	2177 (74.7)	738 (25.3)		667 (50.7)	649 (49.3)	
No/unknown	10133 (71.2)	4099 (28.8)		3943 (49.9)	3961 (50.1)	
**Molecular subtypes**			0.003			0.567
Luminal A	10079 (72.1)	3907 (27.9)		3885 (50.3)	3835 (49.7)	
Luminal B	845 (68.8)	383 (31.2)		300 (48.4)	320 (51.6)	
HER2 enriched	284 (66.2)	145 (33.8)		94 (47.7)	103 (52.3)	
Triple Negative	1102 (73.3)	402 (26.7)		331 (48.5)	352 (51.5)	

RT, radiotherapy; PSM, propensity-score matching.

### Kaplan-Meier analysis before and after PSM

**Figure 1:**
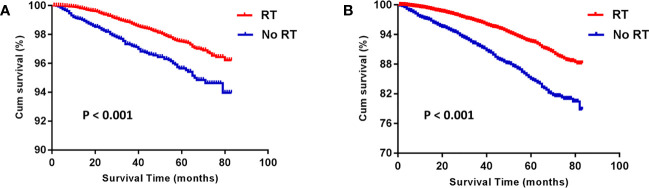
Kaplan-Meier analysis for breast cancer-specific survival (BCSS) **(A)** and overall survival (OS) **(B)** before propensity-score matching. RT, radiotherapy.

**Figure 2:**
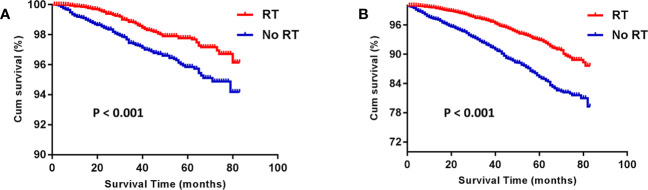
Kaplan-Meier analysis for breast cancer-specific survival (BCSS) **(A)** and overall survival (OS) **(B)** after propensity-score matching. RT, radiotherapy.

Before PSM, the RT group gained an advantage over the No RT group in both BCSS (HR: 1.980 [1.596- 2.456], P < 0.001) and OS (HR: 2.214 [1.966- 2.494], P < 0.001; **Figure 1**). The rates of 5-year survival were 97.5% and 95.5% for the RT and no RT groups, respectively (P < 0.001). After PSM, the similar result was seen in both OS (HR: 2.235 [1.904- 2.624], P < 0.001) and BCSS (HR: 1.918 [1.439- 2.557], P < 0.001) compared the RT group with the No RT group (**Figure 2**). The rates of 5- year survival was 97.7% in the RT group, and 95.4% in the No RT group (P < 0.001).

### Cox regression analysis after PSM

Univariate and multivariate Cox regression analyses was performed after PSM. Based on univariate COX regression analysis results, age, marital status, race, grade, T stage, N stage, RT status, chemotherapy information and molecular subtypes were associated with prognosis of BCSS and OS. In the multivariate COX analysis of BCSS, widowed, divorced and separated patients, tumor grade III, T2 stage, N1 stage, no RT, molecular subtypes with luminal B and triple negative were associated with a shorter BCSS compared with the references (P < 0.05). In the multivariate COX analysis of OS, age ≥74 years, widowed, divorced and separated patients, tumor grade II/III, T2 stage, no RT, no chemotherapy, molecular subtypes with triple negative were associated with a shorter OS compared with the references (P < 0.05). Detailed results are shown in **Table 2**.

**Table 2:** Prognostic factors for BCSS and OS for patients using COX regression analysis.

Variables	BCSS				OS			
	Univariate		Multivariate		Univariate		Multivariate	
	HR (95% CI)	P value	HR (95% CI)	P value	HR (95% CI)	P value	HR (95% CI)	P value
Age								
<74	Ref.		Ref.		Ref.		Ref.	
≥74	1.108 (0.836-1.467)	0.475	1.326(0.994-1.768)	0.055	1.490 (1.271-1.748)	< 0.001	1.500 (1.276-1.764)	< 0.001
Marital status								
Married/Partner	Ref.		Ref.		Ref.		Ref.	
Single	0.846 (0.472-1.518)	0.575	0.893 (0.493-1.616)	0.708	0.918 (0.667-1.263)	0.598	0.900 (0.652-1.244)	0.525
Widowed/ Divorced/ Separated	1.509 (1.127-2.022)	0.006	1.428 (1.060-1.923)	0.019	1.646 (1.399-1.936)	< 0.001	1.533 (1.300-1.807)	< 0.001
Unknown	0.721(0.333-1.560)	0.407	0.832 (0.383-1.807)	0.642	1.121 (0.784-1.603)	0.531	1.089 (0.760-1.558)	0.643
Race								
White	Ref.		Ref.		Ref.		Ref.	
Black	1.640 (1.061-2.537)	0.026	1.079 (0.688-1.692)	0.740	1.474 (1.150-1.890)	0.002	1.213 (0.939-1.566)	0.139
Others	0.854 (0.464-1.573)	0.613	1.059 (0.574-1.953)	0.855	0.684 (0.474-0.988)	0.043	0.738 (0.511-1.067)	0.106
Grade								
I	Ref.		Ref.		Ref.		Ref.	
II	2.437 (1.533-3.874)	< 0.001	1.593 (0.988-2.568)	0.056	1.511 (1.246-1.833)	< 0.001	1.258 (1.031-1.535)	0.024
III	7.942(5.093-12.384)	< 0.001	2.325 (1.357-3.982)	0.002	2.420 (1.969-2.974)	< 0.001	1.592 (1.232-2.055)	< 0.001
Unknown	2.955 (0.890-9.814)	0.077	1.926 (0.576-6.442)	0.287	1.958 (1.113-3.447)	0.020	1.652 (0.936-2.915)	0.083
Laterality								
Right	Ref.		Ref.		Ref.		Ref.	
Left	1.107 (0.838-1.463)	0.473	1.055 (0.797-1.394)	0.710	1.079 (0.927-1.257)	0.324	1.051 (0.902-1.224)	0.525
T stage								
T1	Ref.		Ref.		Ref.		Ref.	
T2	5.121 (3.881-6.758)	< 0.001	2.715 (1.985-3.714)	< 0.001	2.372 (2.012-2.797)	< 0.001	1.965 (1.633-2.364)	< 0.001
N stage								
N0	Ref.		Ref.		Ref.		Ref.	
N1	3.319 (2.429-4.535)	< 0.001	1.931 (1.376-2.710)	< 0.001	1.601 (1.296-1.979)	< 0.001	1.249 (0.995-1.567)	0.055
Radiation record								
Yes	Ref.		Ref.		Ref.		Ref.	
No	1.918 (1.439-2.557)	< 0.001	1.869 (1.402-2.491)	< 0.001	2.235 (1.904-2.624)	< 0.001	2.213 (1.885-2.598)	< 0.001
Chemotherapy								
Yes	Ref.		Ref.		Ref.		Ref.	
No/unknown	0.238(0.179-0.316)	< 0.001	0.824 (0.577-1.178)	0.289	0.708 (0.581-0.862)	0.001	1.282 (1.006-1.634)	0.044
Molecular subtypes								
Luminal A	Ref.		Ref.		Ref.		Ref.	
Luminal B	2.984 (1.917 -4.644)	< 0.001	1.833 (1.139-2.951)	0.013	1.414 (1.066-1.877)	0.016	1.234 (0.914-1.665)	0.169
HER2 enriched	3.448 (1.746-6.806)	< 0.001	1.734 (0.829-3.628)	0.144	1.154 (0.679-1.963)	0.597	0.928 (0.530-1.626)	0.794
Triple Negative	6.365 (4.634-8.742)	< 0.001	3.163 (2.094-4.778)	< 0.001	2.211 (1.778-2.751)	< 0.001	1.691 (1.291-2.214)	< 0.001

RT, radiotherapy; BCSS, breast cancer-specific survival; OS, overall survival; Ref, reference; HR hazard ratio.

### Subgroup analysis for BCSS and OS after PSM

**Figure 3:**
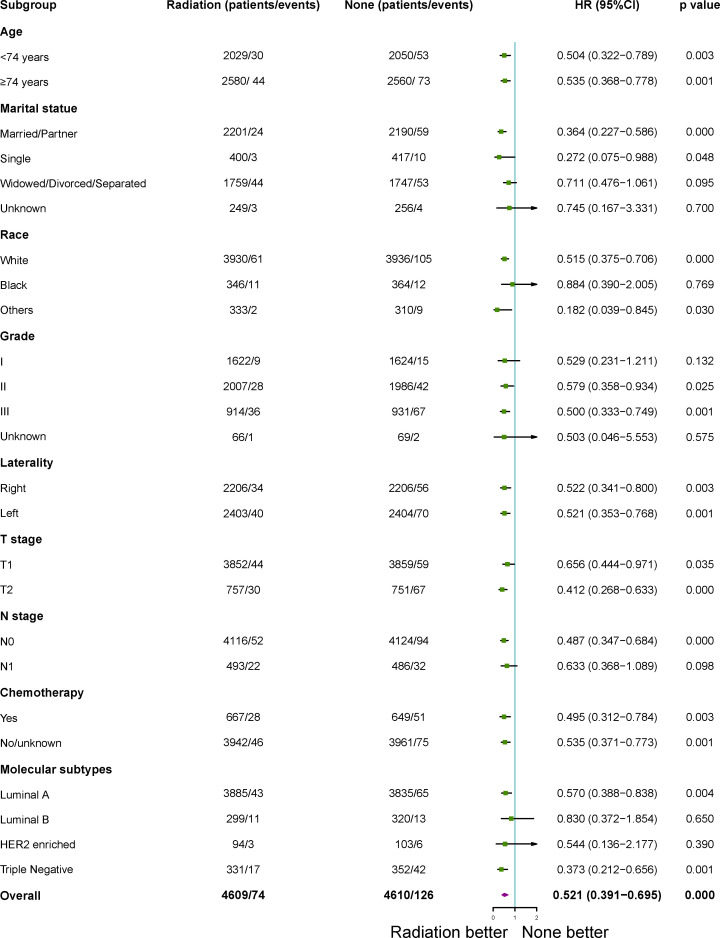
Subgroup analysis for breast cancer- specific survival (BCSS) between adjuvant RT and no RT groups after propensity-score matching. RT, radiotherapy.

**Figure 4:**
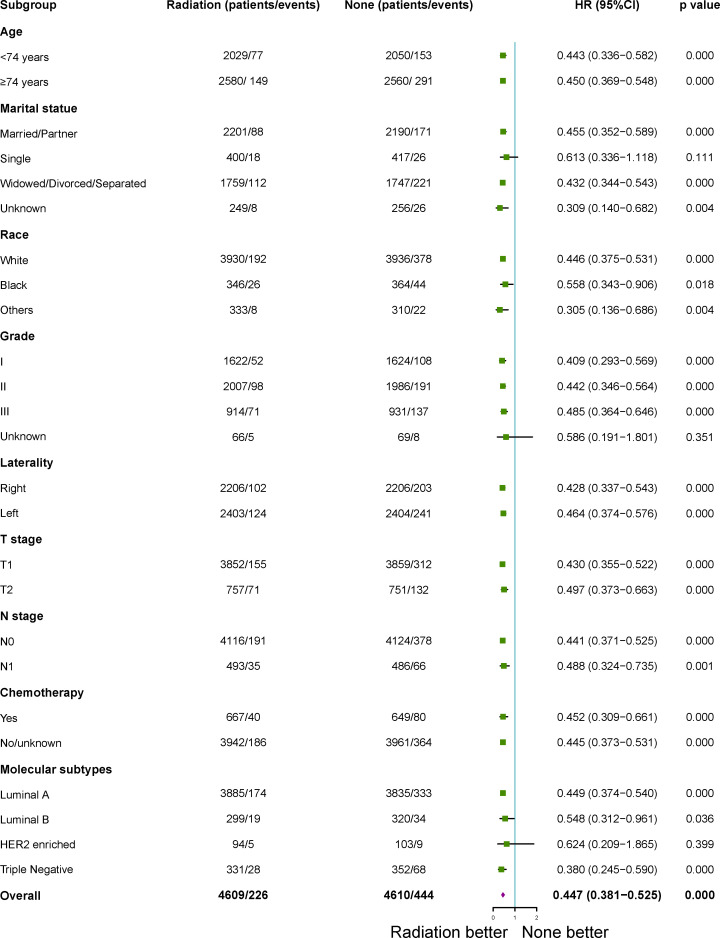
Subgroup analysis for overall survival (OS) between adjuvant RT and no RT groups after propensity-score matching. RT, radiotherapy.

After 1:1 PSM, patients who treated with RT with a higher BCSS and OSwhen compared to those patients who did not in the subgroup analysis. All subgroups analysis-derived BCSS and OS were in support of RT, as observed in the overall study cohort (**Figures 3**, **4**). No matter what age, married/partner patients, single patients, white patients, tumor grade II/III, laterality (left, right), T1-2 stage, N0 stage, chemotherapy (yes, no), molecular subtypes with luminal A and triple negative were statistically in support of RT in terms of BCSS. On the other side, no matter what age, married/partner patients, widowed/divorced/separated patients, white/black patients, tumor grade I/II/III, laterality (left, right), T1-2 stage, N0-1 stage, chemotherapy (yes, no), molecular subtypes with luminal A, luminal B and triple negative were statistically in support of RT, in terms of OS.

## Discussion

Adjuvant radiotherapy after breast-conserving surgery can achieve clear survival benefits in early breast cancer patients (3). However, in elderly female patients, due to the reduced risk of local recurrence, the benefit of RT after BCS may decrease with age (8). In addition, Elderly patients have their own characteristics, often suffering from various underlying diseases and decline in physical function, and the increase in the incidence of postoperative complications, all limit the choice of treatment decisions (9). Furthermore, in many clinical practices, in order to prevent the occurrence of side effects in elderly patients, adjuvant therapy is carried out to the minimum (10). Therefore, the absolute benefits of RT are often overlooked.

Our study retrospectively compared the influence of undergoing adjuvant RT on the prognosis of elderly patients with BCS, to those who had not receive RT. To reduce the impact of unbalanced baseline characteristics, the PSM method was conducted to the data processing. The propensity score distributions and standardized mean differences before and after PSM indicated the balancing covariates between the groups. The results showed that adjuvant RT gained advantages in the management of elderly patients with BCS in both before and after PSM. In the multivariate COX analysis, we observed that adjuvant RT was an independently significant prognostic factor for both BCSS and OS. Additionally, the advantages of adjuvant RT are pronounced in all subgroups of BCSS and OS in the subgroup analysis.

The role of RT in patients with early-stage BC undergoing breast conservation therapy had been established in a number of randomized trials, and most trials had demonstrated local control benefits (2, 4, 5, 11). Blamey et al. (11) reported the incidence of local recurrence after BCS for early breast cancer without adjuvant therapy, regardless of neither RT or tamoxifen, is unacceptably high even with tumors of excellent prognosis. In addition, 10-year follow-up results of the Austrian Breast and Colorectal Cancer Study Group 8A trial suggested patients with hormonal receptor-positive, tumor sizes up to 3 cm, T1 or T2 (early) tumor stages; negative lymph nodes BC treated with BCS and consecutive ET with whole breast RT had a better local control (97.5% vs 92.4%, p = 0.004) and disease-free survival (94.5% vs 88.4%, p = 0.0156) compared with ET alone. The omission of whole breast irradiation (HR: 0.27, p < 0.01) and tumor grade (HR: 3.76, p = 0.03) were the only negative predictors for local recurrence free survival (2). Unfortunately, no further OS benefit was seen.

There have also been some studies suggesting that RT may be associated with better overall survival (8, 12, 13). The Early Breast Cancer Trialists’ Collaborative Group (EBCTCG) meta-analysis suggested a decrease in breast cancer-related mortality and risk of recurrence in both N0 and N1 disease (8). A set of data from Ontario Cancer Registry showed patients with age ≥ 65 years, positive hormonal receptors, stage I BC receive BCS followed by adjuvant RT + ET, adjuvant RT alone, adjuvant ET alone, or no adjuvant treatment. 5 years follow-up results suggested local recurrence rate were 0.9%, 1.4%, 3.1%, 9.4% in adjuvant RT + ET, adjuvant RT alone, adjuvant ET alone, and no adjuvant treatment, respectively (*p* < 0.001). the 5-year overall survival rates were 95.7%, 92.8%, 81.6%, 71.5% in adjuvant RT + ET, adjuvant RT alone, adjuvant ET alone, and no adjuvant therapy, respectively (*p* < 0.001). The rates of BC-related mortality were 0.12%, 1.7%, 1.4%, 1.7% in adjuvant RT + ET, adjuvant RT alone, adjuvant ET alone, and no adjuvant therapy, respectively (*p* = 0.05) (12). This study indicated that RT alone or in combination may still be contributed to overall survival in adjuvant therapy. Additionally, Chu et al. also suggested that Patients who performed a lumpectomy and ET with age ≥70 years, clinical stage I, negative margins, and estrogen receptor positive had better survival outcomes compared those who received RT than those who did not in the 5-year overall survival (87.2% for RT vs 79.4% for no RT, *P* < 0.001) (13).

The CALGB-9343 and RRIME II study had deep impact on clinical practice guidelines. Lacking more detailed data on tumor grading had become a criticism to the trials, which might miss a group of patients who might have benefited from RT (4, 5). Smith et al. found that tumor grading had an impact on the recurrent rate. Among patients who did not undergo RT, the 10-year risk of subsequent mastectomy with high-grade tumors due to recurrence was significantly higher than that of patients with low- to intermediate-grade tumors (11.2% vs 5.5%) (14). In our study, we observed that RT take effect in all tumor grades. In subgroup analysis, patients with grade II (moderately differentiated) and grade III (poorly differentiated) tumors treated with RT yielded better outcome than those did not receive RT in both BCSS and OS.

Although our study included all patients with T2 and N1, regardless of hormonal-receptor status and tumor grade, we did observe that patients would benefit from radiation therapy in all subgroup analysis after PSM. These findings were surprising to contradict previous result. A Prospective, Randomized, Multicenter Trial in Italy with aged 55- 75 years, tumor up to 2.5 cm, 0–3 positive axillary lymph nodes, regardless of hormonal-receptor status and tumor grade, were randomly assigned to BCS + RT or BCS alone. 10 years medium follow-up showed there were no statistically significant differences in in-breast-recurrences and mortality between the two groups (15). In addition, it also can be seen from our research that the response of luminal A type and luminal B type to RT is slightly different, luminal A type to RT has better survival both BCSS and OS, luminal B type to RT has a better survival on OS, but not on BCSS. We didn’t see that in the previous clinical trials and it is suggested that for elderly patients with hormone receptor positive, appropriate treatment methods can be selected according to the type of luminal.

Unlike some important international studies (4, 5), our study performed subgroup analysis and appears to support the use of adjuvant RT in elderly patients with early-stage BC treated with BCS. A decent explanation for our observation is that patients with a better performance status had a much higher chance to receive RT than no RT. Additionally, those in the RT group may have had more opportunities to be timely reminded of the significance of ET, and receiving RT can be used as a safety valve to correct the error of non-compliance to antihormonal therapy.

The omission of radiotherapy also partly comes from the use of third-generation aromatase inhibitors in patients with ER+ BC and targeted therapy in BC patients with HER2 positive status (16). In addition, the side effects, high cost of RT and long treatment time made patients’ compliance poor. Although studies have suggested that RT can cause radiation-induced heart damage, aggravate cardiopulmonary diseases (17), and affect the aesthetics of postoperative breasts (18), such as acute radiation-induced skin reactions and advanced local skin fibrosis. However, in the modern era, hypofractionated RT with higher biological dose saves time cost and makes RT process more convenient for the patients and cheaper payments without compromising outcomes (19). The vast majority of patients reported that the acute severity and long-term side effects of adverse reactions are better or as expected (20).

Several limitations existed in our study. This is a retrospective study. Although we use the PSM method, there is still a chance of bias compared with prospective studies. Specific data on heart and lung function, performance status, nutritional status, comorbidities and major morbidity were not available. Further analysis on the details of induction therapy and chemotherapy regimens were not taken. Additionally, the information regarding lymph vascular invasion, resection margin status, RT dose, RT related field were unknown. The information of salvage therapy with tumor recurrence or progression is lacking.

## Conclusion

We studied whether RT after BCS in elderly patients with early-stage BC should be ignored. Although our inclusion criteria are slightly different from previous clinical trials, we did observe that adjuvant RT may improve patient survival in both BCSS and OS before and after PSM. Furthermore, all subgroups analysis-derived BCSS and OS were in support of RT. Thus, we look forward to further clinical trials to support our conclusions.

## Data availability statement

The raw data supporting the conclusions of this article will be made available by the authors, without undue reservation.

## Ethics statement

The SEER database is a public database and does not require the ethical approval requirements of the institutional review board.

## Author contributions

(I) Conception and design: TH and JH; (II) Administrative support: TH and JH; (III) Provision of study materials: TH, MS and QC; (IV) Collection and assembly of data: TH, MS, QC and DC; (V) Data analysis and interpretation: TH, MS, QC and DC; (VI) Manuscript writing: All authors; (VII) Final approval of manuscript: All authors.

## Supplementary material

Supplementary Figure 1 Distribution of propensity score before and after propensity-score matching.

Supplementary Figure 2 Standardized mean differences before and after propensity-score matching.
