# Supplementary figures and images for: Effect of adjuvant radiotherapy after breast-conserving surgery in elder women with early-stage breast cancer: a propensity-score matching analysis
Source: Front Oncol. 2023 Oct 13;13:1012139. doi: 10.3389/fonc.2023.1012139 (PMC10613027; doi:10.3389/fonc.2023.1012139)

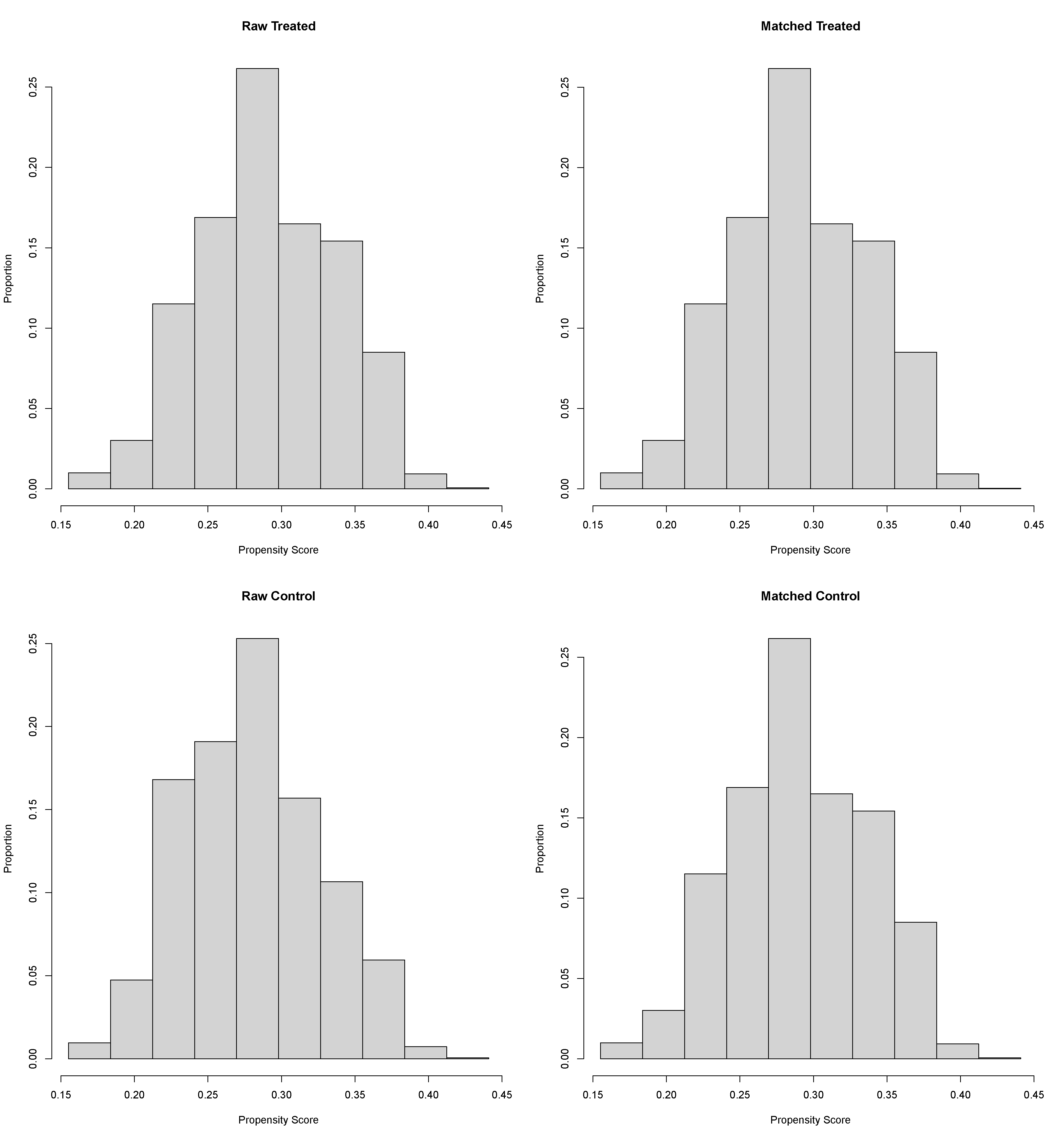

Supplement: Supplementary Figure 1 — Distribution of propensity score before and after propensity-score matching. [file Image_1.tif]

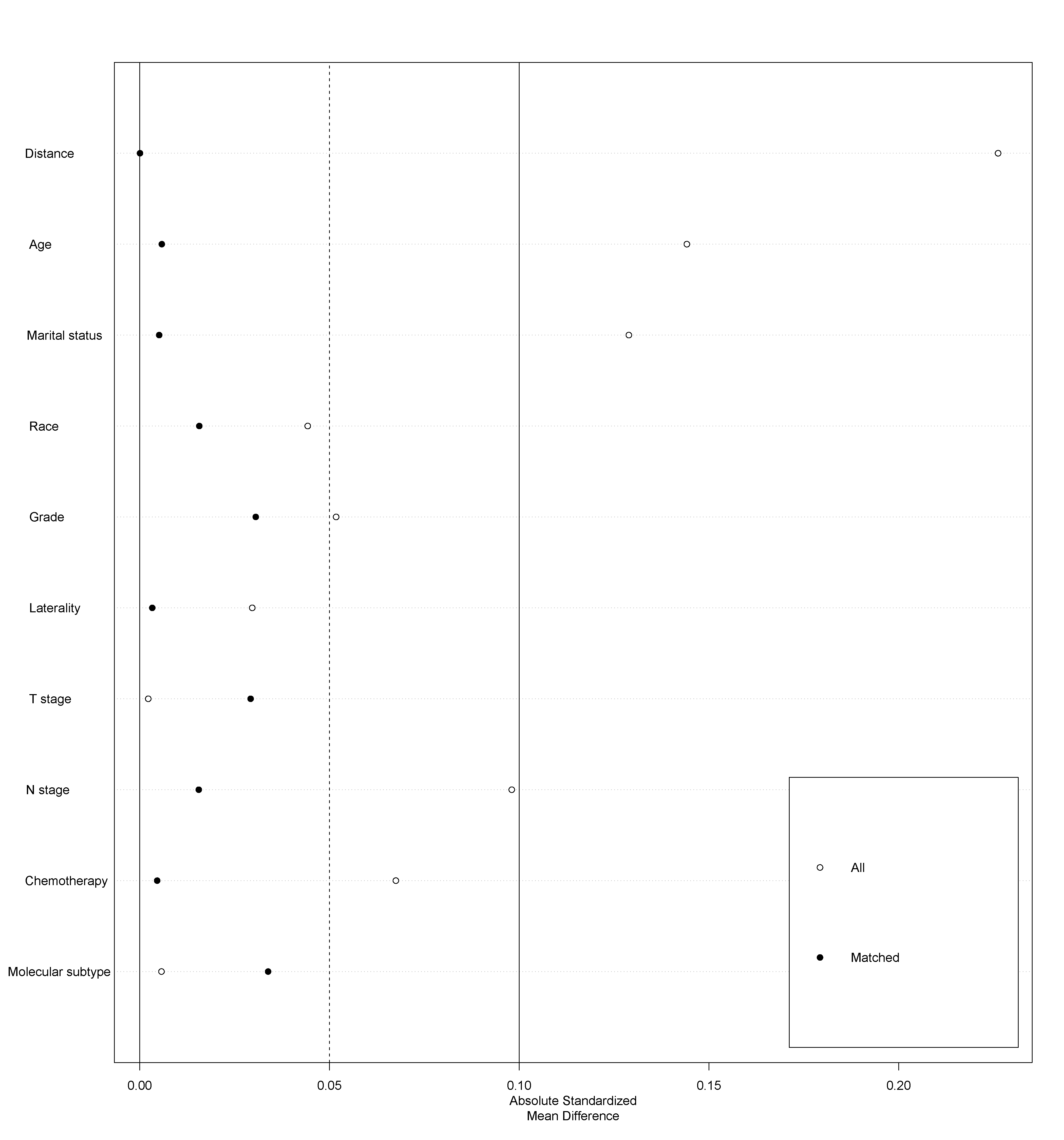

Supplement: Supplementary Figure 2 — Standardized mean differences before and after propensity-score matching. [file Image_2.tif]
